# Are Namibian “Fairy Circles” the Consequence of Self-Organizing Spatial Vegetation Patterning?

**DOI:** 10.1371/journal.pone.0070876

**Published:** 2013-08-15

**Authors:** Michael D. Cramer, Nichole N. Barger

**Affiliations:** 1 Department of Biological Sciences, University of Cape Town, Cape Town, South Africa; 2 Department of Ecology and Evolutionary Biology, University of Colorado at Boulder, Boulder, Colorado, United States of America; Helmholtz Centre for Environmental Research – UFZ, Germany

## Abstract

Causes of over-dispersed barren “fairy circles” that are often surrounded by *ca.* 0.5 m tall peripheral grasses in a matrix of shorter (*ca.* 0.2 m tall) grasses in Namibian grasslands remain mysterious. It was hypothesized that the fairy circles are the consequence of self-organizing spatial vegetation patterning arising from resource competition and facilitation. We examined the edaphic properties of fairy circles and variation in fairy circle size, density and landscape occupancy (% land surface) with edaphic properties and water availability at a local scale (<50 km) and with climate and vegetation characteristics at a regional scale. Soil moisture in the barren fairy circles declines from the center towards the periphery and is inversely correlated with soil organic carbon, possibly indicating that the peripheral grass roots access soil moisture that persists into the dry season within fairy circles. Fairy circle landscape occupancy is negatively correlated with precipitation and soil [N], consistent with fairy circles being the product of resource-competition. Regional fairy circle presence/absence is highly predictable using an empirical model that includes narrow ranges of vegetation biomass, precipitation and temperature seasonality as predictor variables, indicating that fairy circles are likely a climate-dependent emergent phenomenon. This dependence of fairy circle occurrence on climate explains why fairy circles in some locations may appear and disappear over time. Fairy circles are only over-dispersed at high landscape occupancies, indicating that inter-circle competition may determine their spacing. We conclude that fairy circles are likely to be an emergent arid-grassland phenomenon that forms as a consequence of peripheral grass resource-competition and that the consequent barren circle may provide a resource-reservoir essential for the survival of the larger peripheral grasses and provides a habitat for fossicking fauna.

## Introduction

**Figure 1:**
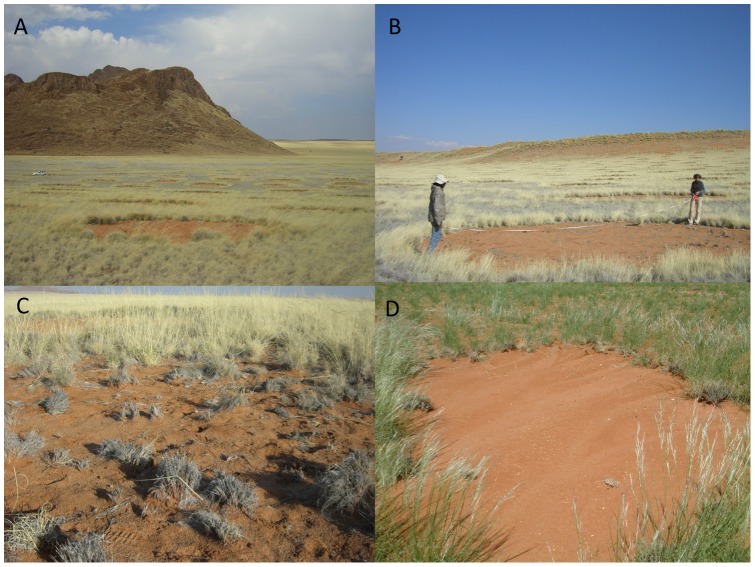
Fairy circles close to “Jagkop” (−24.9770°, 15.8982°) on the NamibRand Nature reserve (June 2012). Site overview (A); a 9.5 m diameter fairy circle being measured (B; the persons in the photograph have given written informed consent, as outlined in the PLOS consent form, to publication of their photograph); grasses do invade the center of some fairy circles, but by this time in the dry season they were mostly dead (C); evidence of surface runoff across a fairy circle following rain (D; photograph by Ann Scott).

Millions of 2–12 m diameter barren “fairy circles” (Fig. 1 A, B) occur in an arid grassland matrix on sandy soils [1] along the eastern edge of the Namib Desert (southern Africa). A ring of peripheral vegetation (commonly *Stipagrostis ciliata* and *S. giessii*) that is taller than the surrounding grassland matrix (commonly *S. obtusa* and *S. uniplumis*) usually surrounds the barren interior [2]. Although this band of taller peripheral grass around fairy circles is common, the rings do also form without the distinctive taller peripheral grasses [3]. The barren fairy circle interior is characterized by higher soil moisture than the matrix soil, probably as a consequence of reduced exploitation of this area by plant roots [4]. The fairy circles are “over-dispersed” with a significantly non-random distribution [5] and are dynamic, appearing and disappearing [2], [6] with a “lifespan” of *ca.* 60 years [3]. Although self-organized over-dispersed pattern formation is common in nature (e.g. sand ripples, dunes, cloud streets), requiring only positive feedbacks [7], this over-dispersed distribution and dynamic nature of fairy circles suggests biogenic causes.

Despite considerable research interest, there is no complete explanation for fairy circles genesis [3]. Causal explanations for fairy circle genesis such as geochemical microseeps [8], [9] are weakened by the fact that this mechanism lacks dynamic and over-dispersed spatial-patterning and that dead vegetation and grass residues may also alter hydrocarbon concentrations in the soil [10], [11]. Although geochemical hydrocarbon microseeps do result in geobotanical anomalies, vegetation change due to these microseeps presents as vegetation stress associated with chlorosis, as opposed to significant plant mortality [12]. Faunal explanations suggest that termites [1], [4], [13] or ants [14] form the fairy circles. Although some reports indicate no correlation between the occurrences of termite nests or belowground foraging tunnels and fairy circles (e.g. [3], [14]–[16]), a recent study showed a strong association between the occurrences of *Psammotermes allocerus* and fairy circles [4]. These termites and the ant *Anoplolepis steingroeveri* are more common on the fairy circles than in the surrounding grassland matrix throughout all life stages of the fairy circle, resulting in the suggestion that, like termite or ant mounds [17], [18], these fauna engineer the fairy circles [4], [14]. Although there is evidence that *P. allocerus* in particular are present early in fairy circle development, there is as yet, no direct evidence that these fauna do indeed engineer the circles (e.g. fairy circle closure upon termite removal).

An alternative hypothesis to those recently proposed is that fairy circles are a self-organizing emergent vegetation spatial pattern [3], [5]. Arid ecosystem vegetation is often discontinuous [19] commonly resulting in vegetation bands, spots or gaps [20]. Patterning may arise from spatial self-organization [21] dependent on the interplay of positive (i.e. facilitative) local-scale (within vegetation band/spot) feedbacks and longer-range (between band/spot and surroundings) negative feedback through competitive interactions [7], [22]–[23]. Recent theoretical modeling [7], [24], [25] confirmed that these patterns could be an emergent phenomenon in arid and semi-arid landscapes. Where such vegetation pattern persists for prolonged periods the soil fertility may be altered, as occurs in soils beneath vegetation canopies that become enriched with nutrients, forming “islands of fertility” [26], resulting in further positive feedbacks to plant growth. Some clonal grasses (e.g. *S. ciliata*) form rings (<0.8 m diameter) with barren interiors [24], [27] which expand outwards [28] as a consequence of water competition, and possibly also autotoxicity between individual ramets [29]. Facilitative feedback results from larger, deep-rooted plants having access to deeper water resources and being able to intercept surface runoff [26], [30]. Resource competition may not only explain ring formation, but spacing of neighboring rings may be related to inter-ring competition for runoff water [7], [21]. Likewise, fairy circle formation has been suggested to result from vegetation patterning and has been mathematically modeled to depend on water availability [31].

**Figure 2:**
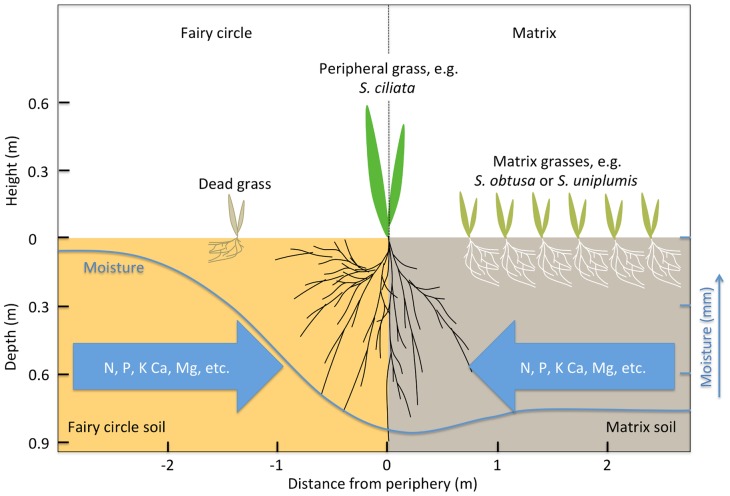
Hypothesized interactions resulting in fairy circle formation. Fairy circles are hypothesized to result from competitive exclusion of grasses on the fairy circles and facilitative access of peripheral species to both fairy circle and matrix water/nutrient resources. Lack of vegetation results in increased soil moisture within the circles. Competition, particularly in surface soils, may compromise grass growth on the fairy circles restricting root development and resulting in death of grasses that do invade the fairy circles. Increased faunal activity due to higher circle soil moisture and lack of propagule establishment may also contribute to maintenance of the barren circles [4], [14]. Arrows show water flux and consequent nutrient mass-flow [32] from the matrix and fairy circle “reservoir” towards deep peripheral grass roots. Nominal sizes of grasses, rooting depths (left axis) and soil moisture variation (blue line, right axis) with distance from fairy circle center are derived from data presented (Fig. 3).

Consequently, our hypotheses for fairy circle development is that these are bare-soil gaps in the otherwise continuous cover of the matrix grasses, as appear in the predictions of theoretical models [7], [31]. We hypothesize that these barren spots arise due to belowground resource-competition between grasses. Enhanced growth in individuals that are strong competitors for belowground resources results in the decline in growth of neighboring individuals, thus forming larger interspaces or barren patches. Water runoff or sub-surface seepage from these barren patches may then move toward nearby plants, which would then further enhance growth. These competitive and facilitative interactions may result in expansion of the vegetation gap or fairy circle as it matures. The vegetation gap or circle results in the creation of a soil water and nutrient reservoir (Fig. 2) from which water facilitates nutrient acquisition by plants [32]. As a consequence, larger grass species may establish on the periphery of the circles, possibly contributing to further expansion of the circles and a water reservoir that persists through the dry season, thus prolonging growth into the dry period. Here we report evidence for competitive and facilitative mechanisms linked to the variation in distribution and morphology of fairy circles.

## Materials and Methods

### Study site and sample collection

Ground survey and soil sampling was carried out at the private NamibRand Nature reserve (−24.9490°, 16.0396°, 1000 m elevation) during June 2012 in the pro-Namib Desert *ca.* 110 km from the coast, between the aeolian Namibian sand sea on the west and Great Namibian Escarpment in the east (Fig. S1). This reserve has been used extensively for research on fairy circles (e.g. [3], [14]). All necessary permits were obtained for the described study, which complied with all relevant regulations (Permit 1698, Ministry of Environment and Tourism; Permit NRNR/P/011/06, NamibRand Nature reserve). The reserve soil is red Kalahari sand with vegetation dominated by *Stipagrostis obtusa* (Dellie) Nees, *S. uniplumis* (Licht) De Winter and *S. ciliata* (Desf.) De Winter of the Poaceae. The average MAP for the area (2006–2011) was 144 mm annum^−1^ and the SD of annual data averaged across sites (n = 19) was 76% of the mean. MAP was also estimated for individual fairy circle sites in this region by using multiple linear regression (MAP  = −63•Latitude +266•Longitude –2501, r^2^  = 0.38, P = 0.029).

Detailed transect-sampling was conducted at 5 sites *ca.* 1–5 km apart (Fig. S1) in which fairy circles were sampled at 4 depths and 1 m intervals from the center of the fairy circle into the matrix. Soils were collected with an auger and depths indicate maximum depth of sample. Soil samples (0–0.3 m depth) were also collected from these and 15 additional fairy circles (distributed *ca.* 1–3 km apart accessed along a circular reserve road) from the center of the fairy circle and the center point between the target fairy circle and the nearest neighbor fairy circle. Soils were immediately double-bagged in heavy plastic in order to maintain field soil moisture. Trenches (0.5 m deep) were also opened from the matrix into the center of the fairy circles and the maximum distance that fine hair-like roots extended into the circles from the peripheral grass stems measured (n = 3). At each of the five sites the areas and distances between fairy circles of 11 nearest neighbor fairy circles were also measured. To compliment these field-based measurements of fairy circle distribution, the Google Earth measurement tool was used to define a 100 x 100 m area for each site. To estimate the degree of over-dispersion in circle spacing, we calculated an “R-value” which is the ratio of actual mean distance between nearest neighbors relative to the expected mean distance based on density. An R-value of <1 indicates clumped distribution, 1 indicates random distribution and 2.15 indicates maximum spacing or maximum dispersion [33].

### Soil analyses

Moisture and bulk density were determined gravimetrically within 3 d of collection by drying soil sub-samples for 48 h at 105°C in a drying oven. Field capacity was measured by wetting soils, allowing them to drain, weighing, drying for 48 h at 105°C in a drying oven and reweighing. Separate sub-samples were dried at 40°C for 36 h and used for nutrient and isotope analysis. The Institute for Plant Production (Department Agriculture: Western Cape, South Africa) conducted the nutrient analyses (pH, resistance, P (citric acid), P (Olsen), K, Mn, Na, Cu, Zn, Ca, Mg) on the unsieved sand (to retain organic material “light fraction”) following standard protocols [34].

Mass spectrometer analysis for N, ∂^13^C and organic C was conducted in the Department of Archeometry (University of Cape Town); *ca*. 40 mg of soil was weighed into tin capsules (Elemental Microanalysis Ltd, Devon, U.K.) and combusted in a Thermo Flash EA 1112 series elemental analyzer and the gasses were fed into a Delta Plus XP isotope ratio mass spectrometer (Thermo Electron Corporation, Milan, Italy). A sub-sample of the soil was also acidified with 1 M HCl for 24 h to remove carbonates and then subject to mass spectrometer analysis for ∂^13^C values of the organic C. The C isotopic ratio of a sample was expressed versus the Pee Dee Belemnite standard. The soil moisture and organic C were summed across the sampled depths using the measured soil bulk density (1738 kg m^−3^).

### Plant cultivation

Wheat (*Triticum aestivum* cv. Baviaans) was germinated in vermiculite and 0.06 m tall plants transplanted (Aug 2010) into pots containing 1.5 kg of sand collected from the top 0.3 m of five fairy circles and five matched matrix sites. Plants were maintained in a temperature-controlled greenhouse (<27°C) at the University of Cape Town, watered daily and moved within the greenhouse thrice weekly to ensure equal exposure to environmental gradients. After 53 d, plants were harvested by carefully washing soil from the roots and separating the plant parts into vegetative shoots, inflorescences and roots and then drying at 80°C for 48 h in a drying oven and weighing.

### Aerial photograph analysis

Using published information as a guide, Google earth imagery for southern Angola and Namibia was searched for fairy circles. For each location a random point cloud was generated in a 50 km radius (Fig. S1). From each of these points terrestrial images were obtained from the Google static map server (maps.googleapis.com/maps/api/staticmap) with the scale  = 1, zoom  = 17, size  = 640×640 and maptype  =  satellite (accessed Sep 2012). These images were then examined to determine whether there were fairy circles evident in the images. This was done conservatively so that only sites with clear fairy circles exhibiting the spatially reoccurring barren patches with clear margins were retained leaving a total of 1 921 images, 80 of which had fairy circles. These presence/absence locations were used for boosted regression tree analysis.

The images for sites with fairy circles were loaded in Matlab (R2012a, The MathWorks, Inc. US), converted to a gray scale, and the fairy circles detected using the “morphological structuring element” tool (strel, with structuring element “disk” and variable operator controlled disk sizes). For each image appropriate parameters were manually set for the number of connected elements, the portion of the image to utilize and which of the recognized fairy circles to discard to obtain good fairy circle recognition by comparison with the color images; problematic cases were discarded. From this image analysis the distances between fairy circles and the density of fairy circles were calculated. These values were found to compare well with those from ground sampling, although the image analysis tended to underestimate the fairy circle area by 11.4%. We did not attempt to correct this because this falls within the precision of ground measurement. These data were used to calculate the R-value [33].

### Boosted regression tree analysis

A machine learning approach involving boosted regression tree (BRT) model construction was performed, as detailed by Elith [35] in R [36]. This procedure yields non-linear models without involving normal null hypothesis significance testing, thus avoiding many auto-correlation problems. Models for fairy circle presence/absence were constructed with (in order of inclusion) the 1^st^ principal component (PC) of the enhanced vegetation index (EVI; spatial-analyst.net; [37]) followed by a subset of the “Bioclim” variables averaged between 1950 and 2000 at 0.042° resolution (www.worldclim.org; [38]), both reprojected to 0.05° using gdalwarp (www.gdal.org). The Bioclim variables were: mean annual precipitation (BIO12), precipitation seasonality (BIO15), mean annual temperature (BIO1), mean diurnal temperature range (BIO2), temperature seasonality (BIO4) and annual temperature range (BIO7). These variables were selected from the 19 Bioclim variable to reduce autocorrelation within the predictor variables. During initial model building, soil pH (H_2_O), cation exchange capacity (clay and fines), total [N], total [C], clay, silt, sand, bulk density and total available water content [39] were also included in the model, but these variables had low explanatory power, and were not retained in the final model. The order of entry of the variables in the model was systematically altered with little effect on the outcome. BRT models were constructed using the ‘gbm’ package version 1.6–3.1 [40] modified by Elith [35] (tree complexity  = 8, learning rate  = 0.0005, bagging fraction  = 0.5 and distribution  =  binomial). After initial BRT analysis, the model was simplified to reduce variance using the recommended procedure [35]. The BRT analysis was used to rank the importance of different predictor variables in determining the fairy circle presence/absence and to search for pairwise interactions of predictors, with all other predictors held at their respective mean values. The full BRT model was used to predict to a spatial grid using the “predict” function in the R package “raster”. The output was converted [37] to a Google earth KML file and the predictions examined.

## Results

### Comparison of fairy circle and matrix edaphic properties

There are relatively few and small differences between characteristics of the soils from the center of the fairy circles and the matched inter-fairy circle matrix soils (Table 1). The major difference is the 2.3-fold higher soil moisture in soil from the center of the fairy circle compared to the matrix soil. The field capacities of the matrix soils were also significantly higher than those of the circles (Table 1). Associated with these differences in water holding capacities are significantly lower soil organic carbon (SOC), soil total [N] and soil [K] and higher available (Olsen) P in the fairy circle than in the matrix soils (Table 1). Other than these small differences in chemical properties, there are no other significant chemical or textural differences. Overall the soils are extremely nutrient impoverished, especially with regard to total [N], total [P] and available [P] which are lower than equivalent values for the nutrient-impoverished soils of the Cape Floristic Region [41]. The ∂^13^C value of the SOC was −14.2±0.3 (Table 1) indicating that SOC was derived from the dominant C_4_ grasses [42] in the region.

**Table 1:** Comparison of soil properties (<0.3 m depth) sampled within the NamibRand Nature reserve between the centers of fairy circles and the center points in the matrix between adjacent fairy circles.

		Unit	Fairy circle	Matrix	P
Moisture	Moisture	%	2.2±0.2	0.95±0.18	**<0.001**
	Field capa city	%	21.7±0.7	18.8±0.8	**0.005**
Chemistry	pH (KCl)		6.72±0.21	6.62±0.2	0.538
	Resistance	Ohms	3795±244	3666±217	0.538
	Organic C	%	0.037±0.003	0.052±0.002	**0.001**
	N	%	0.006±0	0.008±0	**0.001**
	P (citric acid)	mg kg^−1^	10.8±2.3	16±5.9	0.758
	P (Olsen)	mg kg^−1^	1.82±0.18	1.45±0.16	**0.038**
	K	mg kg^−1^	69±4	84±2	**0.003**
	Mn	mg kg^−1^	23.8±2.3	22.4±2.2	0.936
	Na	mg kg^−1^	8.36±0.51	8.27±0.38	0.875
	Cu	mg kg^−1^	0.2±0.01	0.19±0.01	0.67
	Zn	mg kg^−1^	0.16±0.02	0.19±0.04	0.413
	Ca	cmol(^+^) kg^−1^	1.73±0.13	1.73±0.15	0.976
	Mg	cmol(^+^) kg^−1^	1.1±0.04	1.34±0.21	0.259
Texture	Coarse sand	%	32.5±2.4	34.3±3	0.48
	Medium sand	%	9.55±0.95	9.36±1.01	0.69
	Fine sand	%	55±2	54±2	0.639
	Clay	%	1.45±0.16	1.36±0.15	0.676
	Silt	%	1.36±0.20	1.00±0.00	0.104
Isotope	∂^15^N	‰	7.66±0.24	7.19±0.24	0.092
	Organic C ∂^13^C	‰	−14.2±0.3	−13.9±0.1	0.486

Values are mean ± SE (n = 11 sites) and the P values from Student's t tests are indicated in bold where significant.

Wheat was used as a “phytometer” to assess the effects of the soil properties on plant growth. Wheat grew slowly on the soils from both the fairy circles and the matrix (Table 2). However, overall biomass accumulation was 1.4-fold greater on matrix soils than on fairy circle soils. Despite the fact that root growth was reduced more than shoot growth, there were no significant differences in shoot:root ratios.

**Table 2:** Biomass (g dry weight) of wheat grown for 53 d in surface soil (<0.3 m depth) collected from the center of fairy circles or the matrix.

	Fairy circle	Matrix	P
Shoot	0.34±0.02	0.43±0.03	**0.049**
Root	0.28±0.03	0.47±0.05	**0.013**
Inflorescence	0.11±0.01	0.14±0.01	0.111
Total	0.73±0.05	1.04±0.09	**0.020**

Values are mean ± SE (n = 5) and the P values from Student's t-tests are indicated in bold where significant.

### Variation in edaphic properties across fairy circles

**Figure 3:**
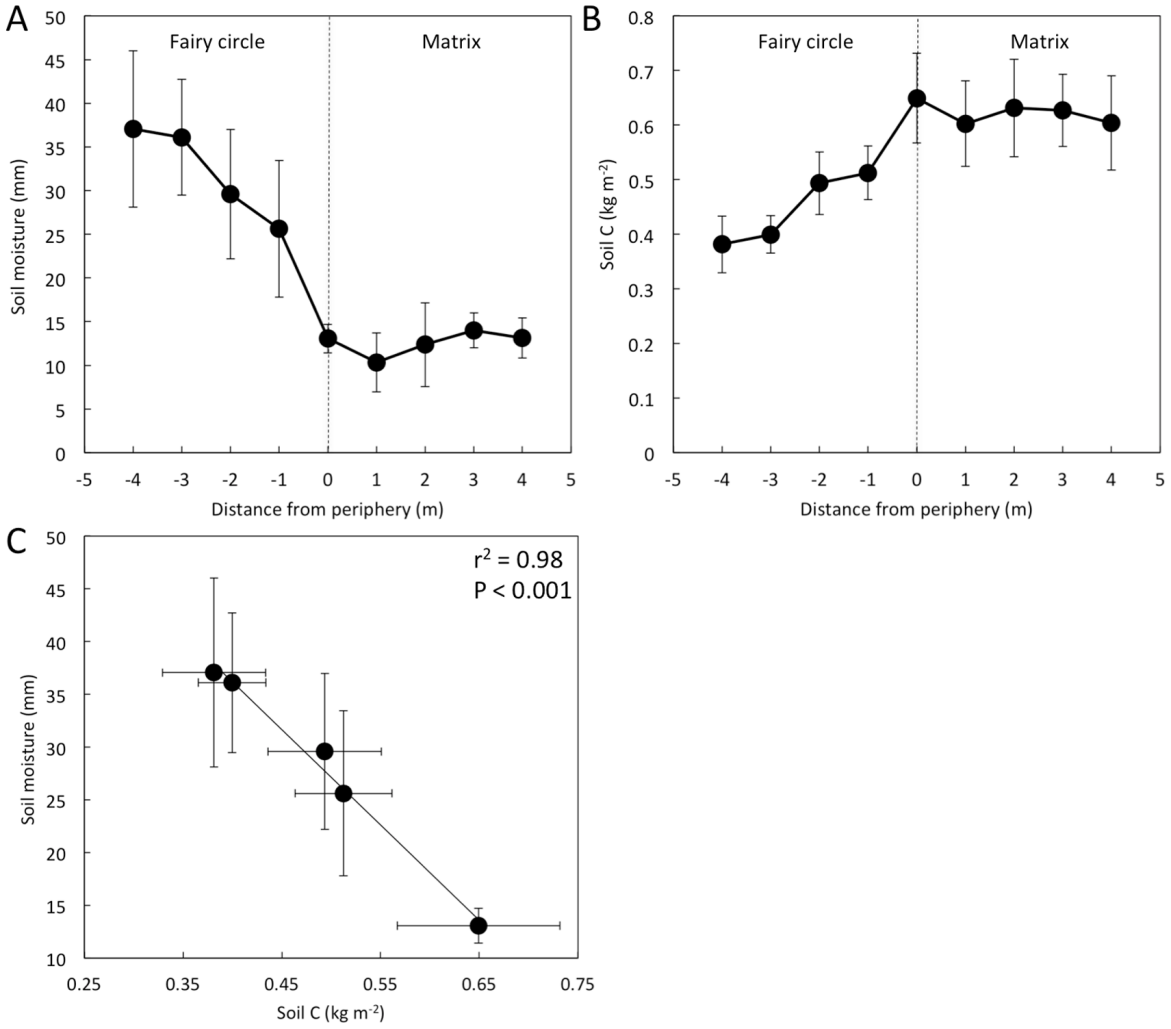
Soil moisture and organic carbon variation across fairy circles and into matrix. Variation with distance from the fairy circle center of soil moisture (A) and organic C (B) weight-summed over sampled depths (mean ± SE; n ≤5). The co-variation of soil moisture and soil organic C (C) is shown with the coefficient of determination (r^2^) and probability value (P) for the subset of data from the barren fairy circle interior.

Within the NamibRand Nature reserve, soil moisture (SM) was highest in the center of the fairy circles (Fig. 3 A). Soil moisture decreased towards the periphery of the fairy circles and was lowest in the soils associated with the matrix. Similar patterns of soil moisture have been reported previously [4], [14]. Soil organic carbon (SOC; Fig. 3 B), which included particulate organic matter (i.e. “light fraction”), also diminished toward the center of the fairy circles and there was an inverse correlation between SM and SOC (Fig. 3 C), possibly indicating that SOC was associated with root biomass that depleted SM. However, coarse roots (i.e. *ca.* 1 mm diameter) were only observed on the side-walls of the excavations within 0.4 m of the plants, whereas sparse finer hair-like roots were observed up to *ca.* 0.9 m from the peripheral plant stems in barren circles.

### Variation of fairy circle morphologies with local edaphic and climate variables

**Figure 4:**
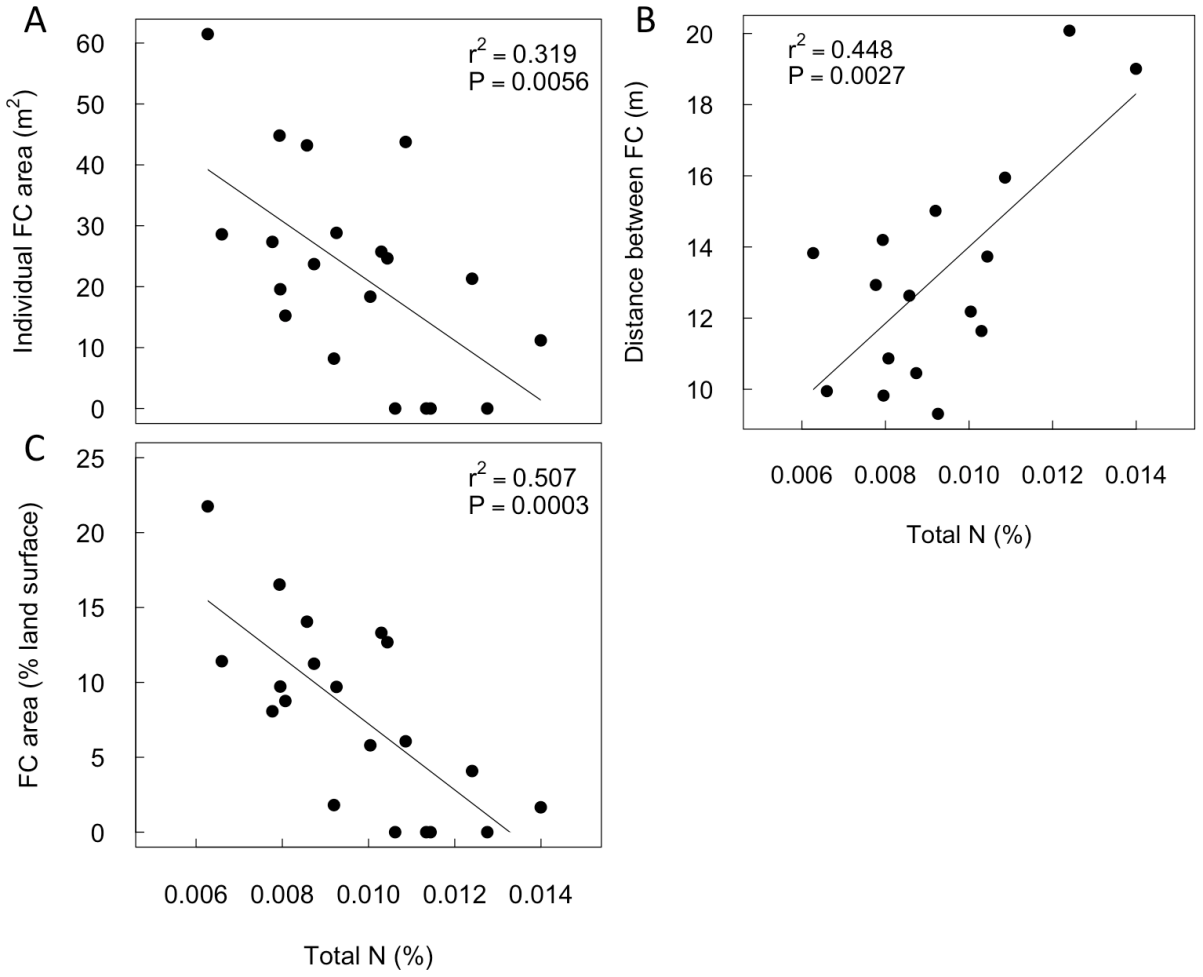
Variation of fairy circle morphological properties with soil total [N] within the NamibRand Nature reserve. Average fairy circle area (A, n = 20), periphery to periphery distances between nearest neighbor fairy circles (B, n = 16), and the fairy circles (FC) landscape occupancy (C, n = 20) were significantly related to matrix soil [N] (<0.3 m depth). Coefficients of determination (r^2^) and probability values (P) are shown. Four sampled sites (Fig. S1) within the reserve had no fairy circles.

**Figure 5:**
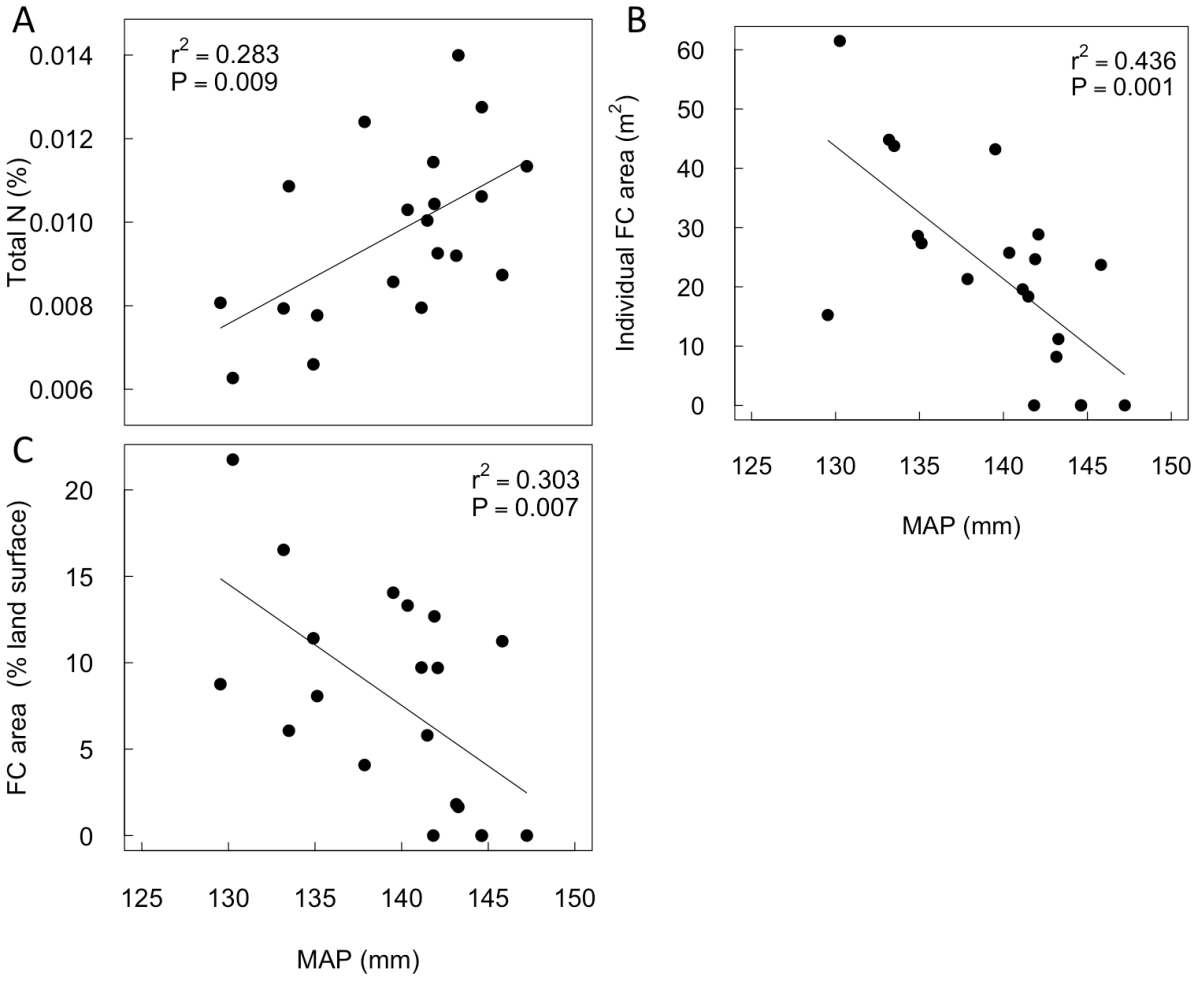
Variation of soil total [N] and fairy circle morphological properties with precipitation within the NamibRand Nature reserve. Site mean annual precipitation (MAP) was estimated from multiple linear regression (latitude and longitude) against data from 19 rainfall collectors in the region. Soil total [N] (A, n = 20), average fairy circle (FC) area (B, n = 16), and landscape occupancy by fairy circles (C, n = 20) were significantly related to MAP. Coefficients of determination (r^2^) and probability values (P) are shown. Four sampled sites (Fig. S1) within the reserve had no fairy circles.

At sites within the NamibRand Nature reserve higher matrix soil total [N] is associated with smaller fairy circles (Fig. 4 A) that are further apart (Fig. 4 B) and have lower landscape occupancy (Fig. 4 C). Other soil variables (e.g. Table 1) were not correlated with fairy circle morphology. Mean annual precipitation (MAP) is positively correlated with matrix soil total [N] (Fig. 5 A), and MAP is inversely correlated with fairy circle size (Fig. 5 B) and landscape occupancy (Fig. 5 C). Individual fairy circle area is also inversely linearly related to matrix SM (Area  = −16.5•SM +63.5, r^2^  = 0.66, P<0.001). The distances between fairy circles were not related to MAP or to matrix SM (data not shown).

### Variation of regional fairy circle occurrence with climate variables

**Figure 6:**
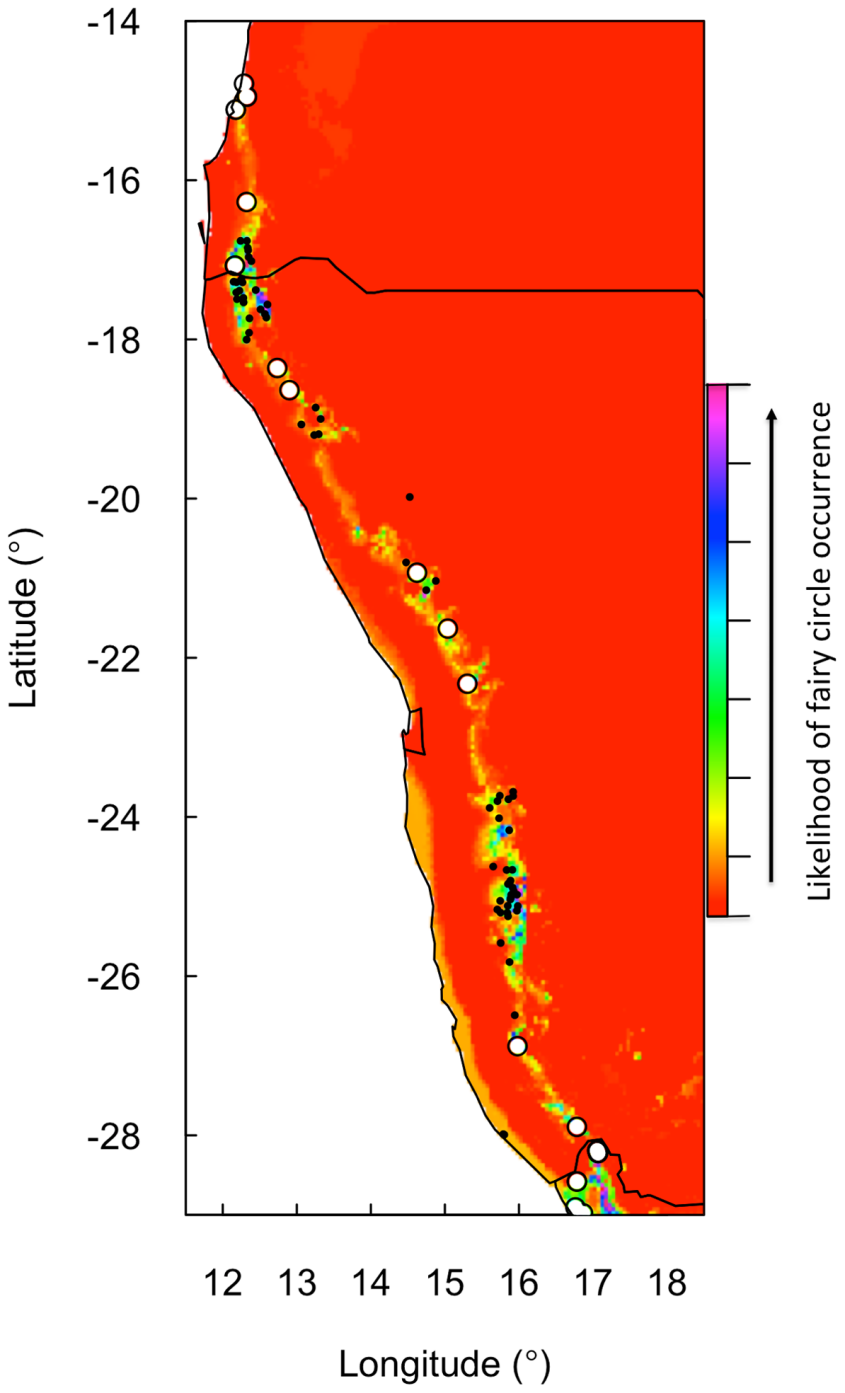
Predictions of fairy circle location likelihood (on an arbitrary scale) from the simplified BRT model (AUC  = 0.95). Black points indicate sites with fairy circles used in model development. The corresponding layer in Google earth (not shown) was used to search for fairy circles outside the ranges of those used in model development. The sites where additional fairy circles were discovered are marked with open symbols. The Namibian borders are shown.

The complete final BRT model included 7 150 trees with a training data “area under receiver operating characteristic” (ROC) curve (AUC) score of 0.95 and a cross-validation AUC score of 0.88±0.01. The AUC score evaluates the classification accuracy of the model (maximum AUC  = 1) and shows that the model was highly accurate. Model performance was also tested by randomly withholding 10% of the data and using the model to predict against the withheld data. BRT predictions of withheld data from this reduced model yielded an AUC of 0.93, indicating good classification performance by the reduced BRT model. Simplification of boosted regression tree (BRT) models of fairy circle presence/absence data retained temperature seasonality, MAP and the 1^st^ principal component of the enhanced vegetation index (1^st^ PC of EVI) as explanatory variables from the original subset of Bioclim, EVI and soil variables that were selected (Table 3). The 1^st^ PC of EVI reflects the spatial variation of the “normalized difference vegetation index” (NDVI) and is a measure of vegetation biomass [43]. Despite a strong positive correlation between MAP and the 1^st^ PC of EVI (data not shown) there was an interaction between the two variables in determining fairy circle presence/absence, because fairy circles occur in narrow ranges of both MAP and vegetation biomass relative to the range in the vicinity of the circles (Table 3). Using these three variables, fairy circle presence/absence was predicted to a narrow geographic range (Fig. 6) and suggests that small changes in MAP, and thus grass cover, could be highly influential in determining fairy circle occurrence. This narrow band of fairy circles occurs within a much wider distribution of the component grasses (Fig. S2).

**Table 3:** The relative influences of the three predictor variables that were retained in the simplified BRT model for fairy circle (FC) presence/absence.

Predictor variable	Relative influence (%)	Interaction	Range (FC present)	Range (FC absent)
MAP (mm)	34	1^st^ PC of EVI (66)	52–135	29–324
1^st^ PC of EVI	31	Temperature seasonality (5)	715–1281	593–2067
Temperature seasonality (%)	35	MAP (40)	193–315	193–417

The variables are mean annual precipitation (MAP), the 1^st^ principal component of the enhanced vegetation index (1^st^ PC of EVI) and temperature seasonality (Fig. S3). The relative interaction sizes (in parentheses) for two-way interactions between these variables were calculated using BRT analyses. The ranges are between the 5 and 95 percentiles of sites sampled either with or without fairy circles (Fig. S1).

**Figure 7:**
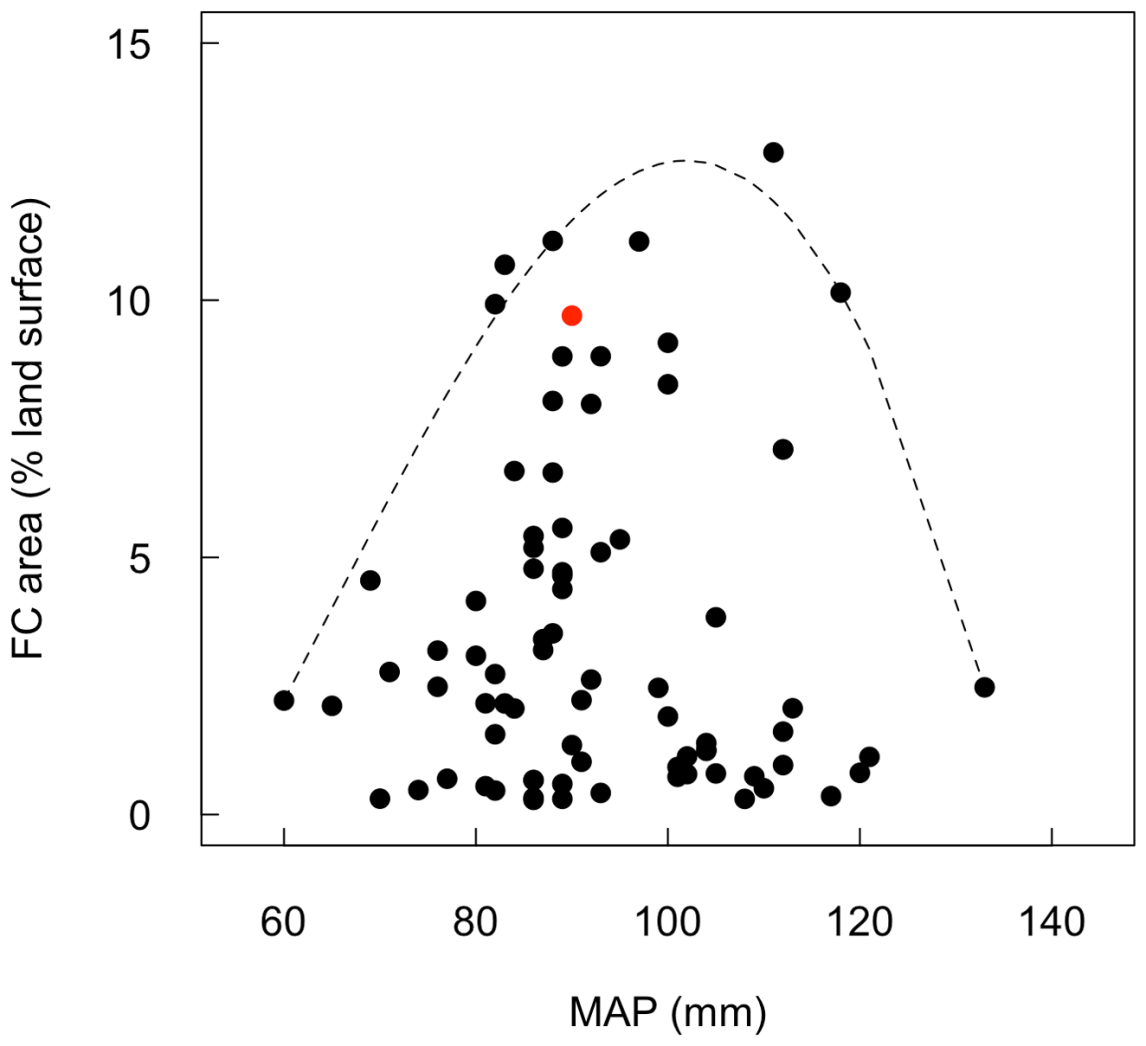
Variation in regional fairy circle (FC) landscape occupancy with mean annual precipitation (MAP). Landscape occupancy was based on aerial photograph analysis (black points) and ground survey (NamibRand Nature reserve site average; red point). MAP was based on Bioclim data (www.worldclim.org; [38]), which is averaged over 1950–2000 and differs from the MAP collected within the reserve (2006–2011). The broken line represents the 90^th^ quantile piecewise non-linear regression line [45] to define the upper limit of landscape occupancy.

There is considerable variation in density, size and distribution of fairy circles over their geographic range (Table 4). Although fairy circles have been reported that exceed 30 m in diameter in Angola [4], we did not assess those sites, and our range of diameters (5 to 95 percentiles, 3.2 to 7.9 m) are more consistent with earlier reports [5]. The range of densities of fairy circles is within those reported previously [4], although the average density for the NamibRand Nature reserve was (mean ± SE, n = 20) 39±4 ha^−1^ compared to previous estimates of 38 ha^−1^ [3] and 56 ha^−1^ [4]. Regionally, fairy circles occupy *ca.* 3.5% of the land surface in areas where they occur, but this ranges up to *ca.* 10%. The distances between the fairy circles (periphery to periphery) are *ca.* 2.8-fold greater than the circle diameters. Unlike the situation within the NamibRand Nature reserve where landscape occupancy of fairy circle is relatively high (mean  = 9.7%) and decreases with MAP (Fig. 4 C), over the full geographic range of fairy circles the landscape occupancy is not linearly related to MAP (Fig. 7). Instead, landscape occupancy is low both in extremely arid and in more mesic sites, and highest in intermediate sites.

**Table 4:** Density, size and distribution characteristics of fairy circles across the established range (Fig. S1, n = 82 sites).

			Percentile	
	Mean ± SE (SE % mean)	Median	5	95
Density (# ha^−1^)	16±2 (10%)	11	2	46
Landscape occupancy (%)	3.5±0.4 (10%)	2.3	0.3	10.2
Area (m^2^)	23±1 (6%)	20	9	52
Diameter (m)	5±0.2 (3%)	4.8	3.2	7.9
Distance between peripheries (m)	14±1 (7%)	11	6	31
R	1.20±0.04 (4%)	1.36	0.58	1.67

Values are the mean ± SE (with the SE as a percent of mean in parentheses), median, and both the 5 and 95 percentiles. The characteristics are the density of fairy circles, fairy circle landscape occupancy (% land surface area), individual fairy circle area, individual fairy circle diameters, the periphery to periphery distances and fairy circle dispersion (R).

**Figure 8:**
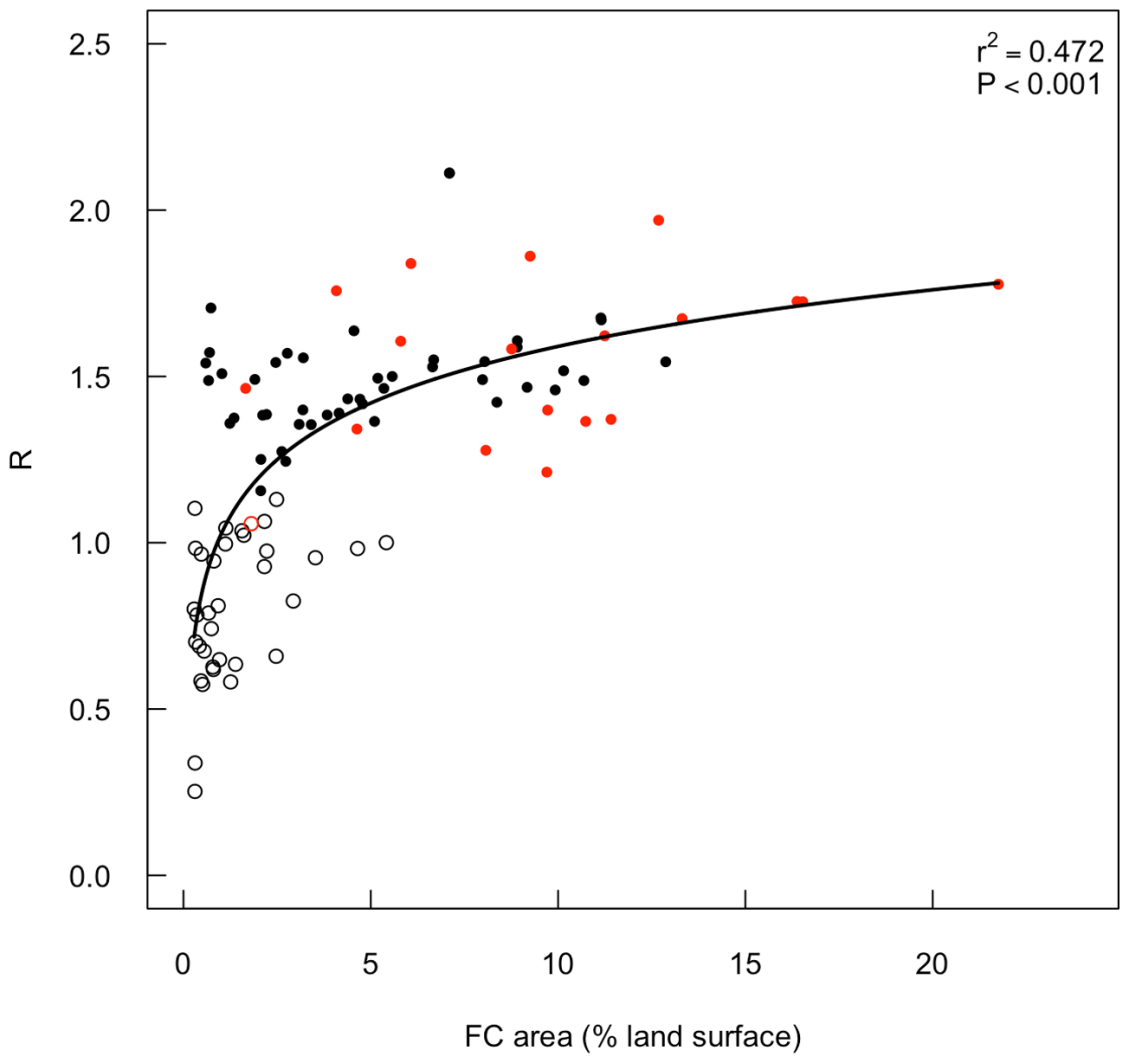
Dispersion of fairy circles. Variation in fairy circle (FC) dispersion (R) with landscape occupancy based on aerial photograph analysis (black points) and ground survey (red points). Closed symbols indicate significantly over-dispersed R-values (Z-test, P<0.05). Fitted line: R = 1.02+0.25Log(FC area). R = 1 for random distributions and 2.15 for maximum dispersion in a hexagonal lattice [33].

**Figure 9:**
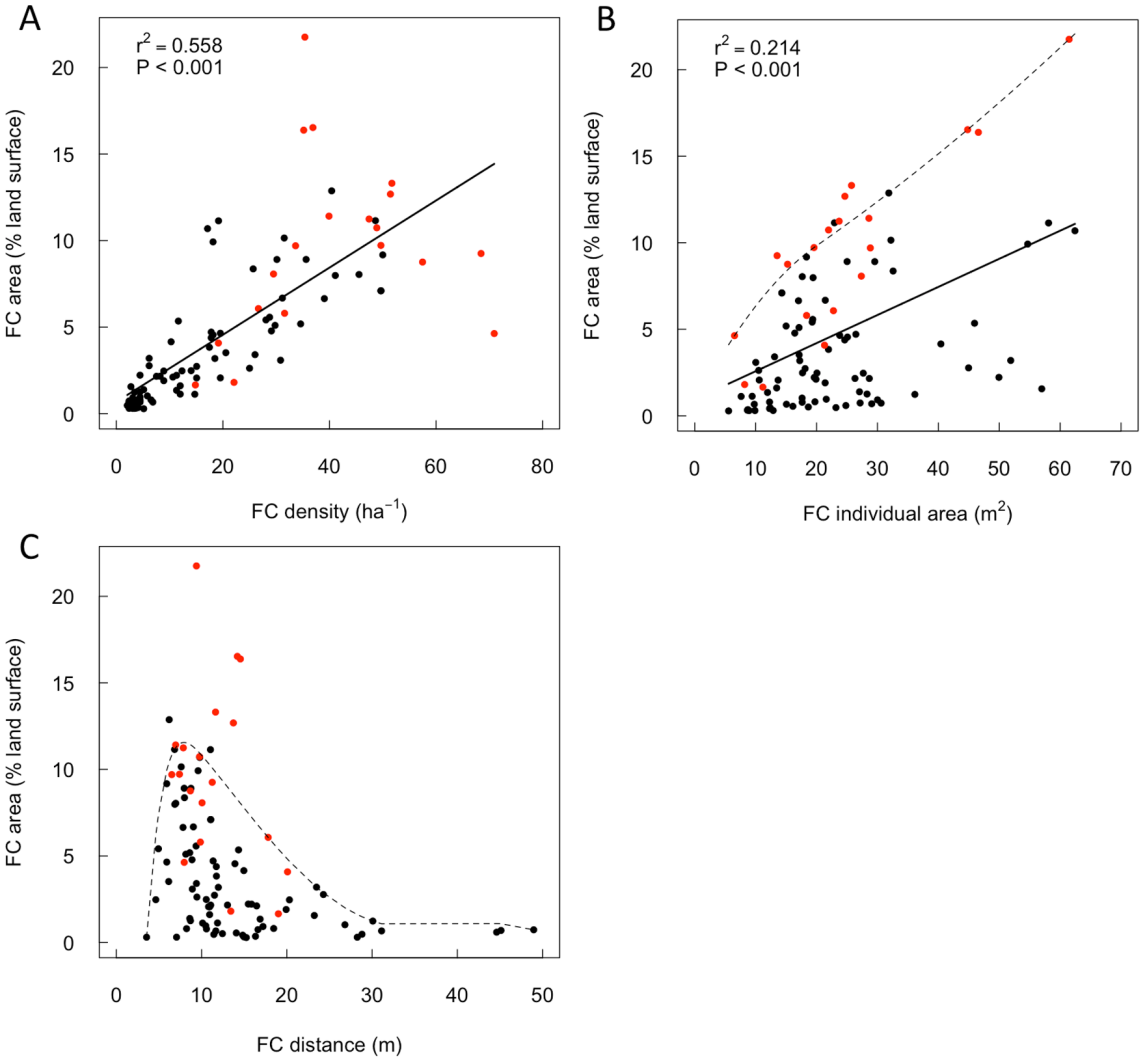
Variation in regional fairy circle (FC) landscape occupancy. Landscape occupancy (FC area as a percentage land surface area) variation with fairy circle density (A), average individual fairy circle area (B) and the spacing between fairy circle peripheries (C). Data is based on aerial photograph analysis of 80 sites across Namibia, and into southern Angola (black points) and sites assessed by ground survey (red points). Coefficients of determination (r^2^) and probability values (P) for regression lines are shown where significant (P<0.05). Broken lines represent 90^th^ quantile piecewise non-linear regression [45] to define the upper limit of landscape occupancy.

**Figure 10:**
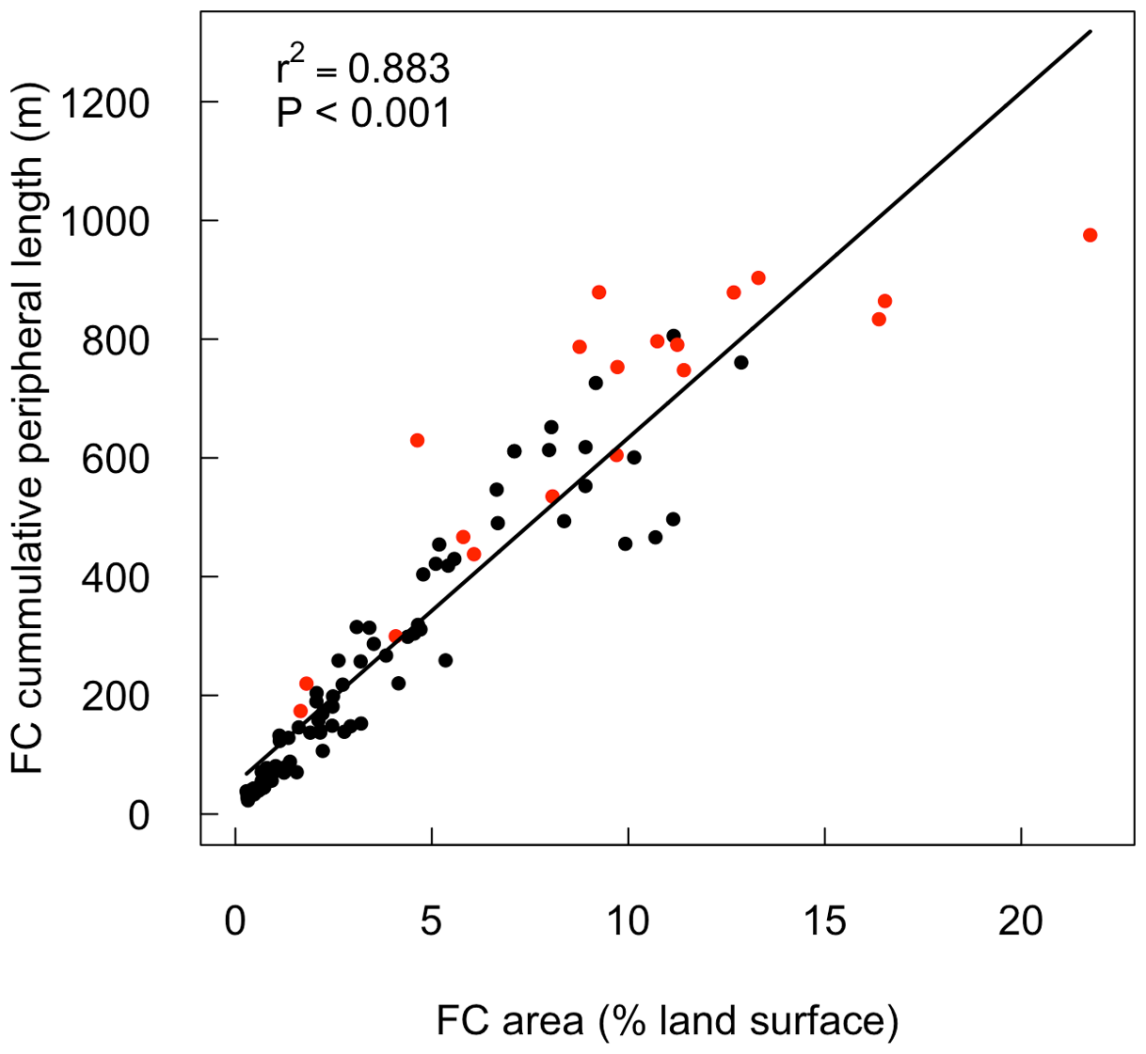
Variation in the cumulative circumference length of fairy circles (FC) per hectare with landscape occupancy of fairy circles. The black points indicate 80 sites across Namibia and into southern Angola measured from aerial photographs. Sites that were assessed by ground survey are shown in red. Coefficients of determination (r^2^) and probability values (P) are shown.

The dispersion (measured with R) of the fairy circles ranges from clumped (R = 0.58) to over-dispersed (R = 1.67) with 58% of sites having statistically over-dispersed fairy circles (Fig. 8). Sites with higher fairy circle landscape occupancies have more over-dispersed fairy circle distributions. Fairy circle landscape occupancy depended on both the density and individual fairy circle sizes, however, density was a stronger predictor of landscape occupancy than individual circle area (Fig. 9 A, B). As expected, fairy circle landscape occupancy was not correlated with the distance between fairy circles, with peak occupancy instead occurring when fairy circles were *ca.* 10 m apart (Fig. 9 C). Cumulative fairy circle periphery lengths were highly correlated with landscape occupancy (Fig. 10). Since *S. ciliata* almost exclusively occurs on fairy circle peripheries in some areas (e.g. NamibRand Nature reserve), fairy circle landscape occupancy may partially indicate the extent of the regional habitat of this species.

## Discussion

We found evidence that both facilitation and competition may be responsible for fairy circles as an emergent vegetation pattern. Within the barren fairy circles, the high SM in the circle centers indicates reduced water consumption there, as concluded previously [4], although canopy interception could also significantly reduce rainfall percolation into matrix soils, particularly of small rain events [46]. The variation of the C_4_-derived SOC across the fairy circles may be due to the distribution of peripheral grass roots that grow inwards from the periphery towards the center of the fairy circle, but do not occupy the centers of the fairy circles. Variation of SOC with depth is commonly strongly associated with root distributions [47] and the presence of roots may also be inferred from depletion of soil moisture [48]. The smooth trajectories of SM and SOC and their inverse correlation suggests that the peripheral grass roots deplete SM, particularly in the surface soils towards the periphery of the fairy circles. In contrast, fine-roots were evident n trenches excavated during the dry season only up to 0.9 m from peripheral plants, with the bulk of root material being within 0.4 m of the plants. While these measures exceed fairy circle peripheral grass root horizontal lengths reported by Juergens (0.2–0.3 m; [4]) this may be because we included fine hair-like roots. Both measures are considerably less than the average of arid-zone perennial grass rooting depths (*ca.* 1.5 m) and maximum horizontal extension (<7.5 m) [49]. Grass roots are fine and difficult to observe in the field, especially during the dry season, resulting in frequent underestimates [50]. SOC may have accumulated and SM been partially depleted during the preceding wet season and indeed indicate root distribution. If roots were, however, only up to 0.9 m from the plant stems, the hydraulic conductivities of sands are high (*ca.* 1 cm min^−1^, [51]) and thus soil volumes adjacent to grass roots that are in hydraulic contact with rhizosphere may serve as a water/nutrient reservoir that buffers seasonal resource-limitations. Since the roots of the peripheral grasses at least partially extend into the fairy circles, consistent with previous work [4], we conclude that the grass roots likely utilize at least part of the fairy circle soil volume that they occupy as a resource, either by direct access or through hydraulic fluxes.

Small fairy circles can form (“births”) and disappear (“deaths”) relatively rapidly (*ca.* 4 years) [3]. Some grasses do invade the barren fairy circles [3], and when successful cause fairy circle “death”, but in most cases these invaders do not persist, as attested by dead tussocks within the fairy circles (Fig. 1 C). Despite being significant, differences in soil nutrients between the fairy circles and the matrix were small, consistent with the fact that others have failed to detect significant differences (e.g. [5], [13]). Thus we do not know whether these small differences between circle and matrix soils occur outside the study area. These differences in the nutrient-impoverished (<0.01% total [N] and <2 mg kg^−1^ available [P]) coarse sands may, however, contribute to the marginality of the fairy circles for plant growth at the study site. Significantly reduced greenhouse growth (50%) of *S. uniplumis* on fairy circle soils relative to matrix soils [52] and similar results with ryegrass [5] and wheat (Table 2) may be due to lower [N] and/or lower field capacities in fairy circle soils (Table 1), although plants grown on fairy circle soils also have lower mycorrhizal infections [52] and the circle soils have also been reported to have lower microbial biomass [53]. Edaphic limitation of growth may also cause reduced root growth of fairy circle-invaders (*ca*. 50% reduction, [14]) and contribute to their mortality. Although some circles may be short-lived, many are stable for several decades [3]. Lower soil [N] and [C] and consequently lower field capacities in fairy circle relative to matrix soils probably result from a combination of a prolonged lack of vegetation in the fairy circles and wind erosion. Importantly, we do not suggest that these small soil differences are the cause of fairy circles, but rather that they are an emergent property of fairy circle longevity that may contribute to maintenance of the barren circles. Long-term maintenance of fairy circles may also be influenced by propagule availability, since wind blown grass diaspores do not readily lodge on the barren fairy circles unless trapped in faunal burrows [14], thus perpetuating the spatial pattern. Furthermore, both the termite *P. allocerus* [4] and the ant *A. steingroeveri* [14] are more abundant on fairy circles than in the matrix, and may contribute to grass mortality within fairy circles. Indeed, dead grass tussocks on some circles frequently have symptoms of termite damage. Thus a complex set of constraints including competition, nutrient-availability, lack of propagules and faunal activity may all contribute to fairy circle maintenance.

The hypothesis that fairy circles are an emergent competition-induced phenomenon is supported by evidence that local-scale resource availability (soil total [N], SM and MAP) is inversely correlated with the variation in fairy circle size and landscape occupancy. The correlation between inter-circle spacing with N, but not MAP, possibly indicates that this spacing is a complex product of these and other variables, requiring further investigation. Because MAP is positively correlated with soil [N], it is not possible here to discern which of these resources is most important in determining fairy circle size and landscape occupancy, although it is likely that both contribute (e.g. [32]). In an arid ecosystem where water is barely sufficient to support plant growth, resource-competition is inevitable, especially when combined with nutrient impoverished soils. The negative effects of resource availability on fairy circle morphology at a local scale are consistent with the regional occurrence of fairy circles. Accurate prediction of fairy circle presence/absence using the simplified BRT model indicates that the few predictors (temperature seasonality, MAP and the 1^st^ PC of EVI) captured important environmental determinants of fairy circle occurrence. The geographically narrow band of fairy circles within the much wider distribution of the component grasses also indicates that fairy circles are an emergent climate- and vegetation-dependent phenomenon.

In this arid area the inter-annual variability of MAP is high and this, combined with the narrow range of MAP over which fairy circles occur, may result in sites readily becoming either too arid or too wet, possibly accounting for the dynamic nature of small fairy circles reported previously [3]. We suggest that sites that are marginal for fairy circle development may rapidly (< *ca.* 4 years) transition between fairy circle presence and absence, depending on rainfall variations. In high precipitation years, we would predict that resource limitation will be relaxed resulting in fairy circle “death” or closure. An additional consideration in fairy circle closure is the presence of faunal activity. In a recent study of grass colonization of fairy circles [4], conditions of high termite activity were associated with low numbers of grasses recolonizing the circle. As termite activity decreases, however, recolonization may occur. Interestingly, however, a significant number of fairy circles that exhibited low termite activity, also showed low recolonization by grasses [4]. Taken together, these patterns suggest that multiple factors are likely to control fairy circle closure. Overall, environmental predictors of fairy circle occurrence are consistent with facilitative/competitive interactions determining fairy circle occurrence. Furthermore, the distribution of fairy circles is consistent with mechanistic model predictions [7], with this “gap vegetation” pattern (i.e. fairy circles) being replaced in more arid sites to the west by “spot vegetation” and subsequently by barren sand.

Sites with high fairy circle landscape occupancy also have more over-dispersed circles (i.e. high R-values), consistent with fairy circles “competing” with each other for resources and resulting in over-dispersed spacing only at high landscape occupancies. Landscape occupancy is correlated with both density and fairy circle areas, as reported previously [3], but in our analysis is more dependent on density than average area of the fairy circles. Cumulative fairy circle periphery lengths increased with fairy circle landscape occupancy. The taller *S. ciliata* occurs almost exclusively on the peripheries of the circles in some areas (e.g. NamibRand Nature reserve). In these situations fairy circle landscape occupancy, and thus cumulative peripheral lengths, may partially be a measure of the niche-space for this species. Thus maximum habitat utilization by the peripheral species in these sites occurs when fairy circle landscape occupancy is maximal, which is when the circles are spaced *ca.*10 m apart (Fig. 9 C).

Competitive spacing of the fairy circles may be related to harvesting of surface runoff, as suggested for grass rings in the Negev [7]. Although surface infiltration rates are high in the Namib [10] and runoff low (<10 mm annum^−1^) [54], runoff does sometimes occur (Fig. 1 D). Surface runoff may thus contribute to fairy circle formation, but whether this fits with mostly circular patches without skewed grass density or shapes regardless of slope, is questionable. One might expect surface runoff to result in down-slope elongation of fairy circles. Unlike *Bouteloua gracilis* grass rings in the Chihuahua desert [30], no aeolian accumulation of sand occurs around fairy circle peripheries that might increase infiltration there. Instead we propose that sub-surface flow of water driven by water gradients setup by deep peripheral grass roots in the coarse sands with high hydraulic conductivities [51] may result in “competition” between fairy circles for resources, resulting in over-dispersed spacing at high landscape occupancy. This sub-surface flow of water may also be pertinent for explaining vegetation patterns elsewhere.

## Conclusions

Both model predictions [31] and the data presented here suggest that fairy circles emerge as a consequence of vegetation spatial patterning, resulting from competition for nutrient- and water-resources and positive facilitative feedbacks associated with the fact that the barren fairy circles provide a resource-reservoir. In some cases fairy circles allow taller peripheral grasses to survive in a matrix of smaller grasses in these resource-impoverished landscapes where they would otherwise be rare. Fairy circles may thus represent an emergent phenomenon in which peripheral grasses, possibly together with fauna (e.g [4], [14]), participate in the construction of their own resource-niche and a faunal habitat in an arid and nutrient-impoverished landscape.

## Supporting Information

Figure S1 **Locations of sample sites.** Map showing sites randomly sampled for fairy circle occurrence (n = 1 921), sites where fairy circles were confirmed to be present (n = 82), and sites sampled by ground survey in the NamibRand Nature reserve (n = 20). A typical image used for estimation of fairy circle areas, spacing and density can be viewed in Google Maps™ (http://goo.gl/K3GXi; accessed 2013-05–10; location −24.981361°, 15.952531°).
(TIF)

Figure S2 **Distribution of southern African collection sites (data from** [**44**] **) of the** ***Stipagrostis*** **species at fairy circle sites.** *S. ciliata* and *S. giessii* form fairy circle peripheries and *S. obtusa* and *S. uniplumis* are common in the matrix. Each species includes all sub-species (not differentiated on figure).
(TIF)

Figure S3 **Geographic variation of environmental variables utilized in BRT model for predicting fairy circle occurrence.** Temperature seasonality (A; SD mean monthly temperatures ×100); 1^st^ principal component of enhanced vegetation index (1^st^ PC of EVI) indicating vegetation biomass (B); mean annual precipitation (C; MAP). The Namibian borders are shown on each map and the points indicate sample location found to have fairy circles.
(TIF)
