# Supplementary figures and images for: Are Namibian “Fairy Circles” the Consequence of Self-Organizing Spatial Vegetation Patterning?
Source: PLoS One. 2013 Aug 15;8(8):e70876. doi: 10.1371/journal.pone.0070876 (PMC3744476; doi:10.1371/journal.pone.0070876)

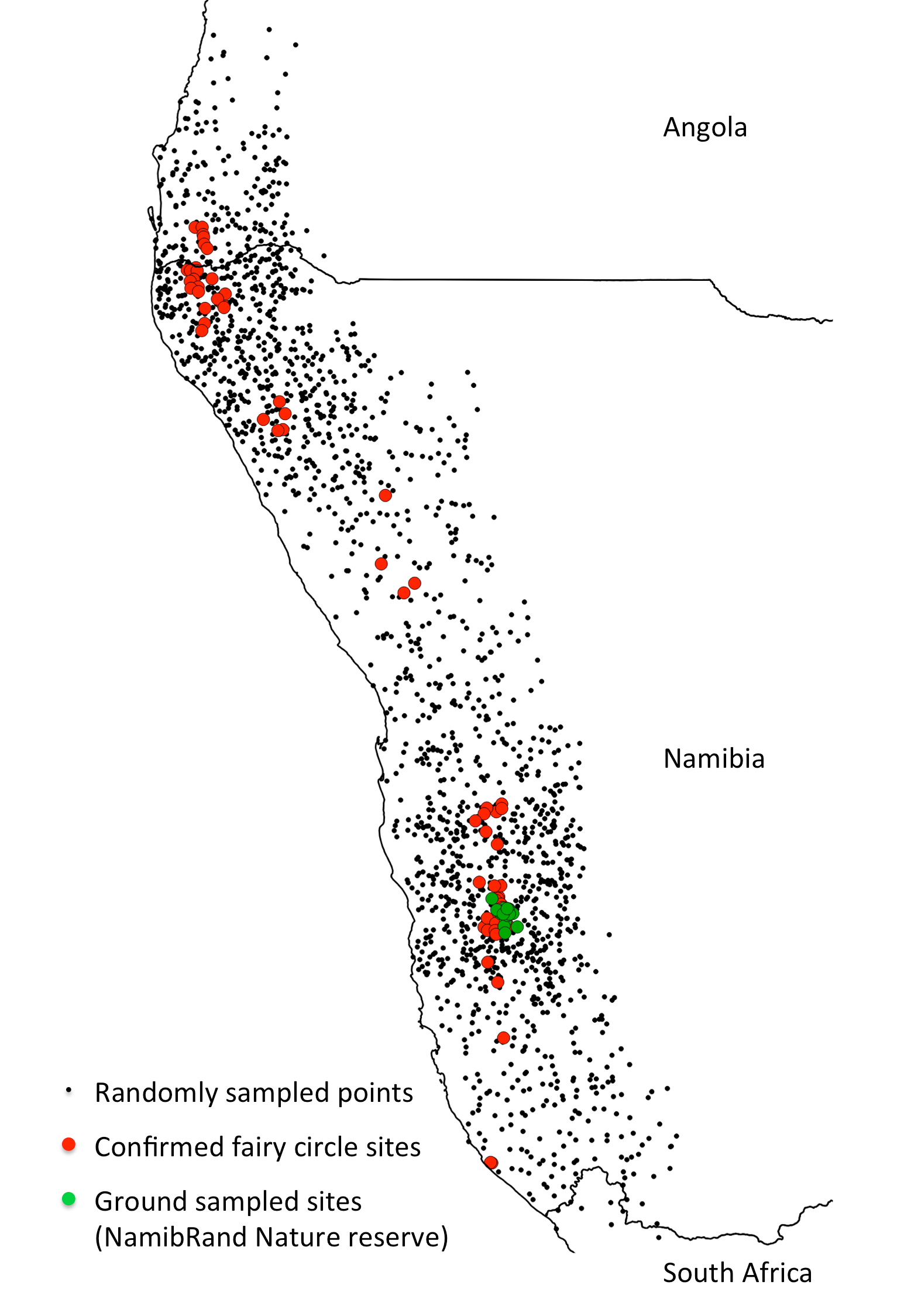

Supplement: Figure S1 — Locations of sample sites. Map showing sites randomly sampled for fairy circle occurrence (n = 1 921), sites where fairy circles were confirmed to be present (n = 82), and sites sampled by ground survey in the NamibRand Nature reserve (n = 20). A typical image used for estimation of fairy circle areas, spacing and density can be viewed in Google Maps™ (http://goo.gl/K3GXi; accessed 2013-05–10; location −24.981361°, 15.952531°). (TIF) [file pone.0070876.s001.tif]

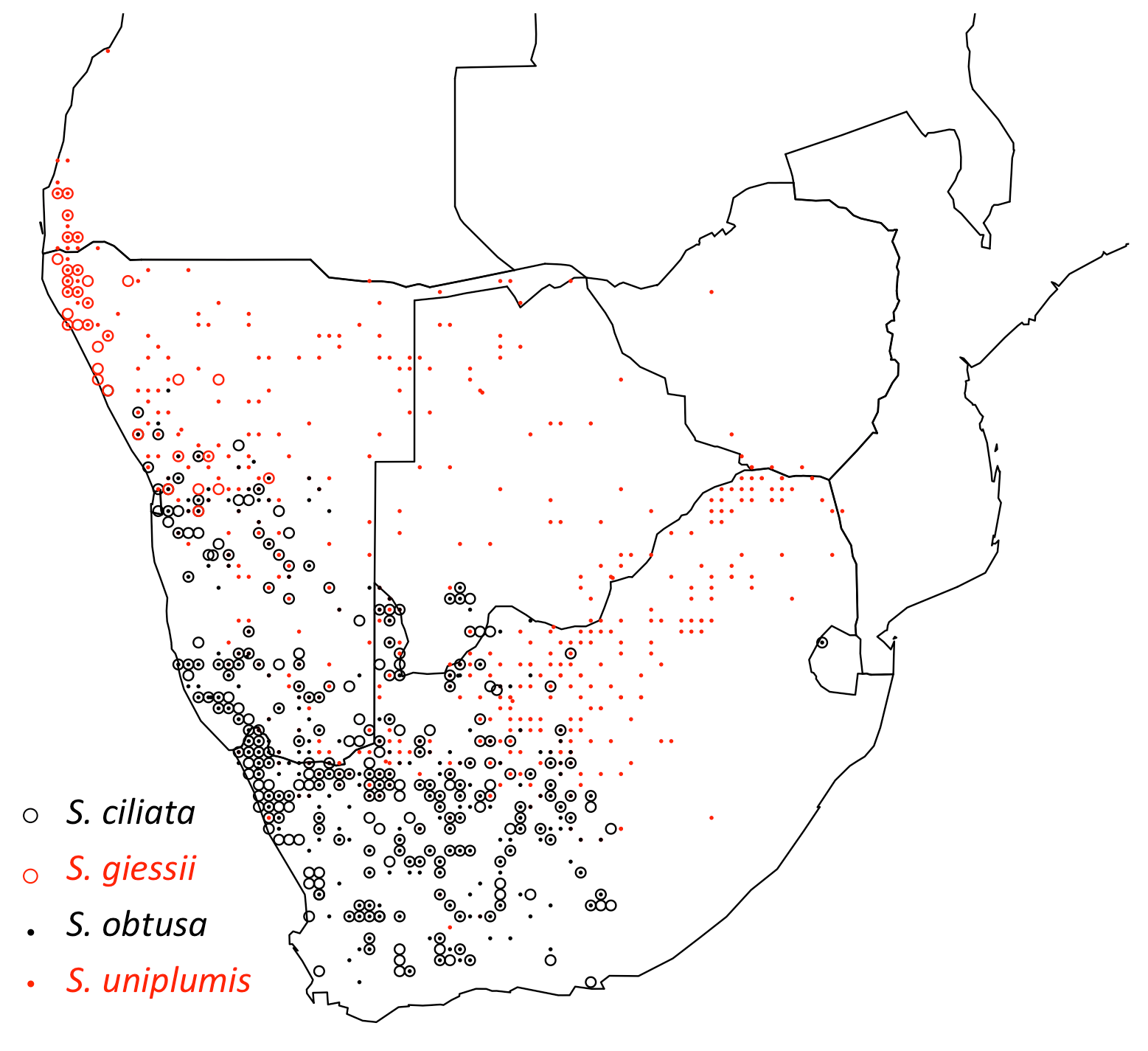

Supplement: Figure S2 — Distribution of southern African collection sites (data from [44] ) of the Stipagrostis species at fairy circle sites. S. ciliata and S. giessii form fairy circle peripheries and S. obtusa and S. uniplumis are common in the matrix. Each species includes all sub-species (not differentiated on figure). (TIF) [file pone.0070876.s002.tif]

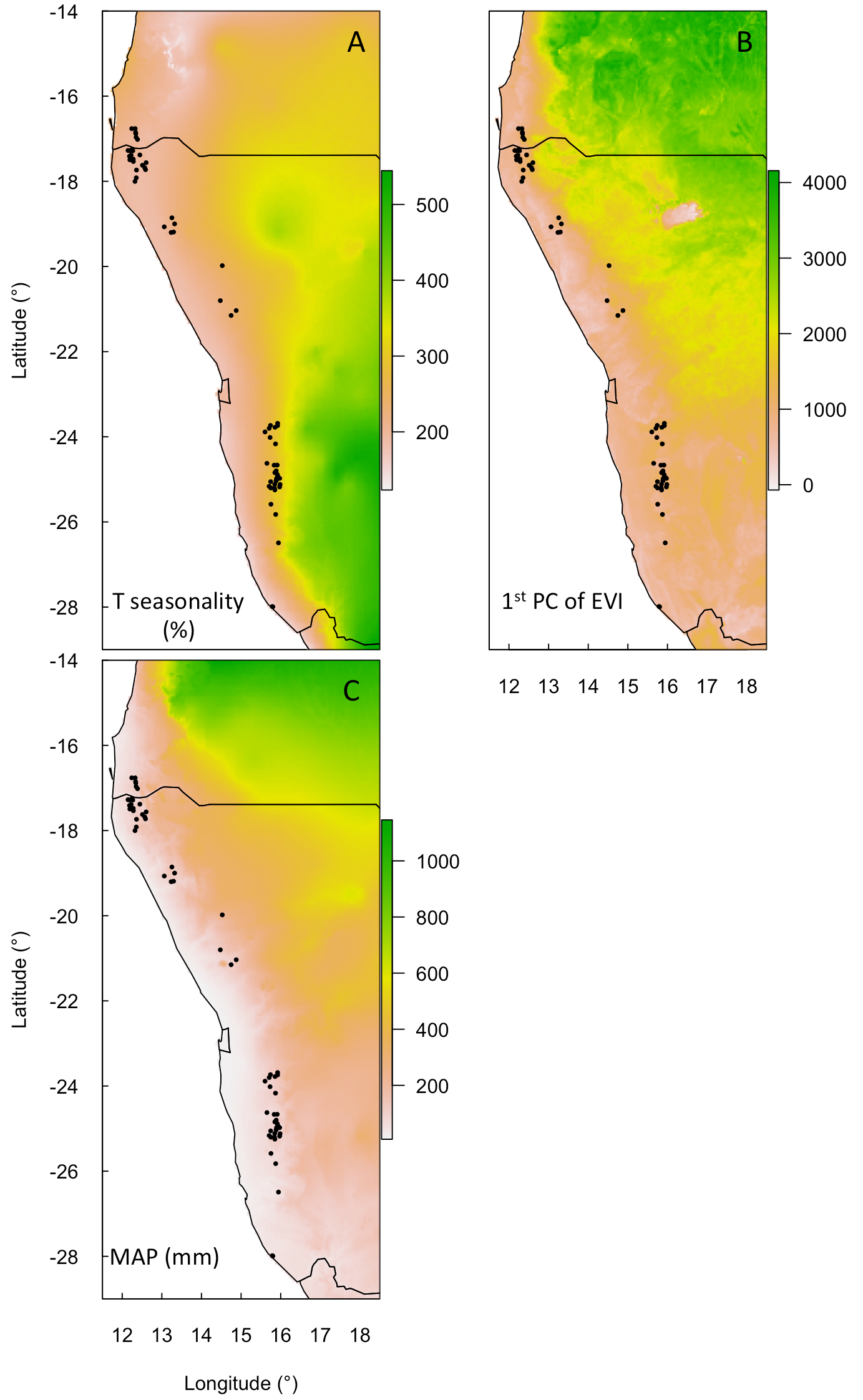

Supplement: Figure S3 — Geographic variation of environmental variables utilized in BRT model for predicting fairy circle occurrence. Temperature seasonality (A; SD mean monthly temperatures ×100); 1st principal component of enhanced vegetation index (1st PC of EVI) indicating vegetation biomass (B); mean annual precipitation (C; MAP). The Namibian borders are shown on each map and the points indicate sample location found to have fairy circles. (TIF) [file pone.0070876.s003.tif]
